# Endoscopic therapy using an endoscopic variceal ligation for minute cancer of the esophagogastric junction complicated with esophageal varices: a case report

**DOI:** 10.1186/1752-1947-4-149

**Published:** 2010-05-23

**Authors:** Tomoyuki Akiyama, Yasunobu Abe, Hiroshi Iida, Hiroki Endo, Kunihiro Hosono, Kyoko Yoneda, Hirokazu Takahashi, Masahiko Inamori, Akihide Ryo, Shoji Yamanaka, Yoshiaki Inayama, Atsushi Nakajima

**Affiliations:** 1 Gastroenterology Division, Yokohama City University School of Medicine, Japan; 2 Department of Pathology, Yokohama City University Hospital, Japan

## Abstract

**Introduction:**

Standard endoscopic mucosal resection or endoscopic submucosal dissection is a procedure for patients with minute cancers, complicated with esophageal varices that puts them at high risk of bleeding.

**Case presentation:**

We present the case of a 77-year-old Japanese man with alcoholic cirrhosis who underwent a routine endoscopy examination as a screening procedure for esophageal varices and was incidentally diagnosed as having minute cancer of the esophagogastric junction with esophageal varices. Endoscopic ultrasonography findings suggested that the minute cancer was a non-invasive carcinoma (carcinoma *in situ*) and a 2 mm in diameter blood vessel, feeding the esophageal varices, pierced the lesion. Following the examination, we carried out endoscopic treatment of the minute cancer and esophageal varices. Endoscopic variceal ligation was performed using a pneumo-activated device (Sumitomo Bakelite, Tokyo, Japan). Two years after the treatment, during the follow-up endoscopic examination on the patient, recurrence of carcinoma was not detected endoscopically or histologically.

**Conclusion:**

Endoscopic therapy using an endoscopic variceal ligation device for minute cancer of the esophagogastric junction, complicated with esophageal varices, may be an acceptable and easily applicable method.

## Introduction

Minute cancer is a gastric cancer lesion of less than 5 mm in its maximum diameter, with tumor cells confined to the mucosa. Lymph node metastases, meanwhile, are extremely rare [1,2]. These characteristics of minute cancer provide the basis for using endoscopic therapy as a curative treatment [3]. In this report, we describe endoscopic therapy using an endoscopic variceal ligation (EVL) device for minute cancer complicated with esophageal varices.

## Case presentation

**Figure 1:**
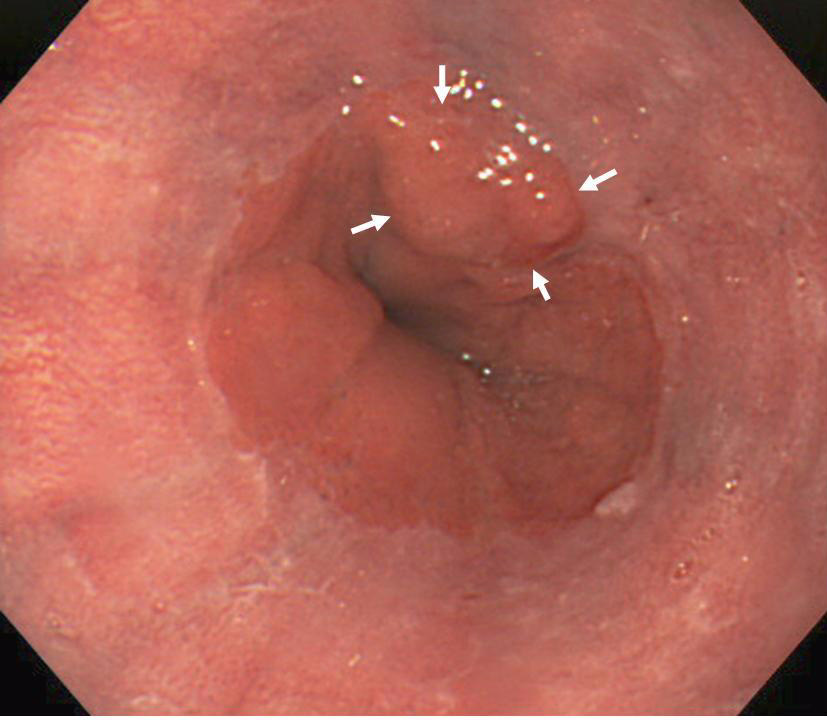
**Minute cancer of the esophagogastric junction**. White arrows indicate the minute cancer.

**Figure 2:**
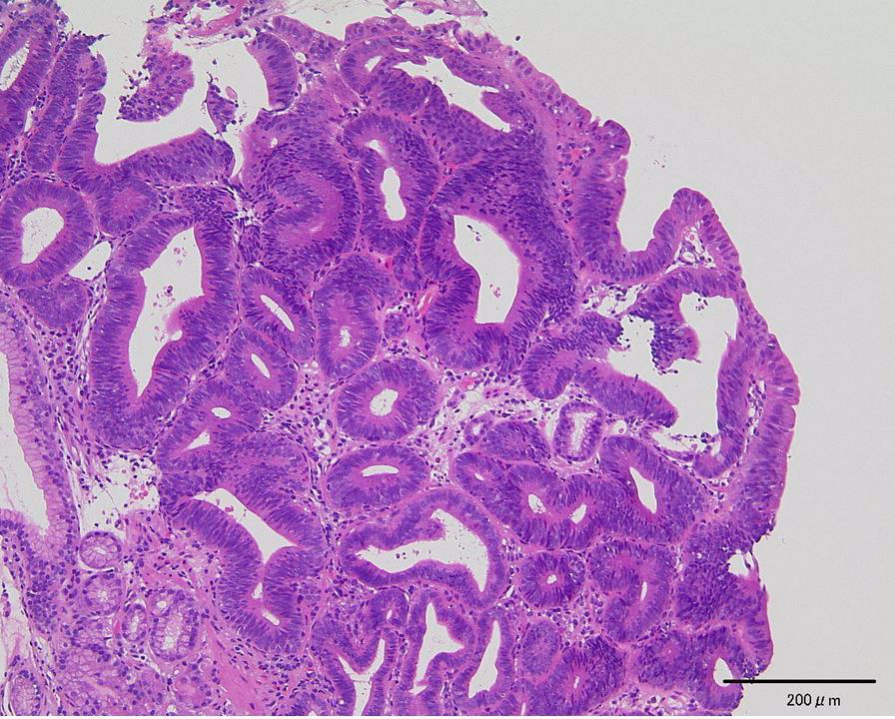
**Histological examination of the biopsy specimen showed well-differentiated adenocarcinoma (hematoxylin and eosin staining, original magnification ×100)**.

**Figure 3:**
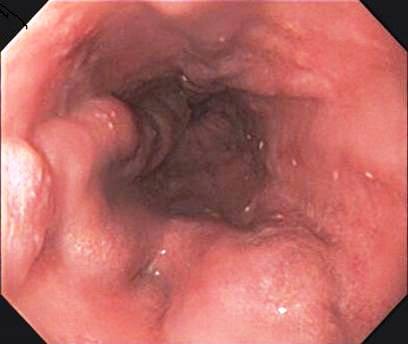
**Esophageal varices**. (A) The lower esophagus and gastroesophageal junction. (B) The middle esophagus.

**Figure 4:**
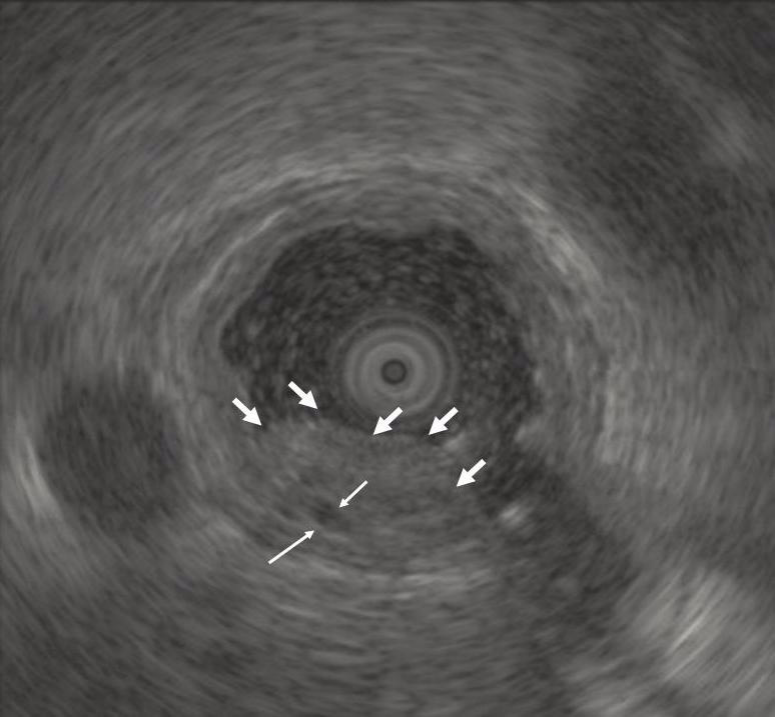
**Endoscopic ultrasonography with Olympus UM2000 (Olympus Optical Company, Tokyo, Japan), demonstrates that the minute cancer was noninvasive carcinoma (carcinoma *in situ*) and a 2-mm diameter blood vessel (thin white arrows), feeding the esophageal varices, pierces the lesion**. Thick white thick arrows indicate the minute cancer.

A 77-year-old Japanese man with alcoholic cirrhosis had been undergoing a follow-up laboratory examination every month for three years. He had been undergoing imaging studies such as ultrasonography and computed tomography every month as screening procedures for hepatocellular carcinoma, and endoscopic examination every year to monitor esophageal varices. During one such routine examination, he was diagnosed as having minute cancer of the esophagogastric junction (EGJ) (Figure 1) based on the histological examination of the biopsy specimen (Figure 2) and esophageal varices (Figure 3). One month later, he was referred to our institution for endoscopic ultrasonography (EUS) evaluation (Olympus UM2000, Olympus Optical Company, Tokyo, Japan) and endoscopic therapy. The ultrasonography findings showed that the minute cancer was a noninvasive carcinoma (carcinoma *in situ*) and a blood vessel, 2 mm in diameter feeding the esophageal varices, pierced the lesion (Figure 4).

Following the EUS examination, we carried out endoscopic treatment of the minute cancer and esophageal varices. The endoscope (GIF-Q200, Olympus Optical Co., Tokyo, Japan) was preloaded with an overtube, which was passed into the oropharynx over the already introduced endoscope. The tip of the endoscope was loaded with a pneumatic EVL device (Sumitomo Bakelite, Tokyo, Japan). Under full endoscopic suction, the minute cancer was tightly packed inside the cap of the endoscope, and the tripwire was pulled, creating an artificial polyp that included the lesion. After confirmation that the electrocautery markings were contained in the ligated band, resection was not performed for the lesion. EVL was successively done for the esophageal varices in three places. All EVL bands were placed in the esophagus and EGJ. The iatrogenic ulcers in the esophagus and EGJ resulting from the EVL were treated with the administration of a proton pump inhibitor and sodium alginate.

**Figure 5:**
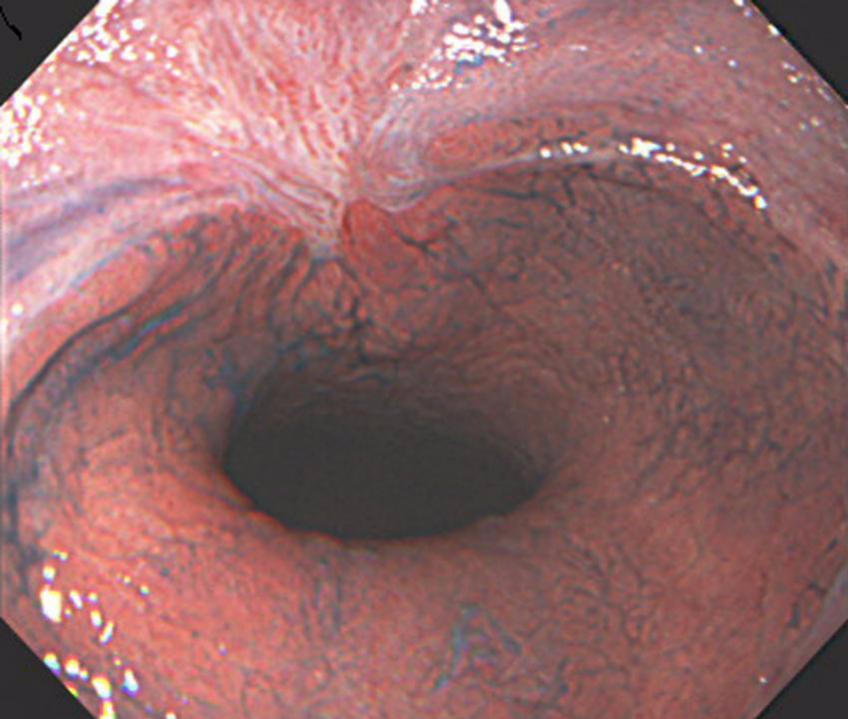
**Iatrogenic ulcer has healed with scarring, but without any residual lesion**.

The ulcers were observed endoscopically three months after the procedure to check for the presence of any residual lesion or other lesions, and forceps biopsy specimens were obtained from the site of the resection. A follow-up examination after two years did not show recurrence of the disease (Figure 5).

## Discussion

Endoscopic resection has the advantage of providing a full specimen for histological examination and for the diagnosis of the extent and infiltration depth of the lesion. The outcome of endoscopic therapy can therefore be evaluated on the basis of macroscopic and histologic findings. However, in our case, our patient with minute cancer, complicated with esophageal varices, was at a high bleeding risk because of standard endoscopic mucosal resection or endoscopic submucosal dissection. We thus selected endoscopic therapy using EVL, without mucosal resection, for this lesion. This technique had several limitations that may need to be considered. First, large lesions cannot be completely excised with this method because of the size limitation of the friction-fit adaptor. In our case, the diameter of the cancer was less than 10 mm in diameter. Second, because a full specimen cannot be provided, histological examination and the diagnosis of the extent and infiltration depth of the lesion cannot be evaluated.

## Conclusion

We believe that because of its technical simplicity and safety, endoscopic therapy using an EVL device for minute cancer of the EGJ, complicated with esophageal varices, may be an acceptable and easily applicable method.

## Abbreviations

EGJ: esophagogastric junction; EVL: endoscopic variceal ligation; EUS: endoscopic ultrasonography.

## Consent

Written informed consent was obtained from our patient for publication of this case report and any accompanying images. A copy of the written consent is available for review by the Editor-in-Chief of this journal.

## Competing interests

The authors declare that they have no competing interests.

## Authors' contributions

TA, YA, HT and KY analyzed and interpreted our patient data. HI, HE and KH analyzed endoscopic data. AR, SY and YI performed the histological examination of the organs. TA, MI and AN were major contributors in writing the manuscript. All authors read and approved the final manuscript.
